# Maintenance Therapy With Avelumab for Patients With Metastatic Urothelial Carcinoma: A Real‐World, Ambispective RAVE‐Bladder Study

**DOI:** 10.1002/cam4.70636

**Published:** 2025-02-14

**Authors:** Ilya Tsimafeyeu, Yana Gridneva, Alexander Sultanbaev, Yulia Anzhiganova, Mark Gluzman, Anastasia Mochalova, Alexey Shkurat, Edgar Israelyan, Elvira Parsadanova, Yulia Murzina, Aleksei Ivanov, Alexey Kalpinskiy, Olesia Stativko, Vladislav Petkau, Elena Karabina, Artyom Kеln, Natalya Tovbik, Sufia Safina, Maria Turganova, Evgeny Kopyltsov, Varvara Bragina, Olga Novikova, Andrei Lebedinets, Vladislav Vodolazskiy, Alexey Rumyantsev, Ruslan Zukov, Ilya Pokataev, Rashida Orlova, Maria Volkova

**Affiliations:** ^1^ Bureau of Cancer Research—Moscow Office Moscow Russian Federation; ^2^ Oncological Center No.1 of Moscow City Hospital Named After S.S. Yudin Moscow Russian Federation; ^3^ Republican Clinical Oncology Dispensary Ufa Russian Federation; ^4^ A.I. Kryzhanovsky Krasnoyarsk Regional Cancer Center Krasnoyarsk Russia; ^5^ Medical Institute Saint‐Petersburg State University Saint‐Petersburg Russian Federation; ^6^ Clinical Hospital #1, Medsi Otradnoe Moscow Russian Federation; ^7^ Department of Medical Oncology SBHI Leningrad Regional Clinical Hospital Saint‐Petersburg Russian Federation; ^8^ N.N. Blokhin National Medical Research Center of Oncology Moscow Russian Federation; ^9^ Sakhalin Regional Clinical Oncology Dispensary Yuzhno‐Sakhalinsk Russian Federation; ^10^ Birobidzhan Regional Cancer Center Birobidzhan Russian Federation; ^11^ Center for Immunotherapy and Targeted Therapy Moscow Russian Federation; ^12^ P.A. Herzen Moscow Oncological Research Institute Moscow Russian Federation; ^13^ Sverdlovskiy Regional Oncological Dispensary Ekaterinburg Russian Federation; ^14^ Tula Regional Oncology Dispensary Tula Russian Federation; ^15^ Department of Oncology, Radiology and Radion Therapy Tyumen State Medical University Tyumen Russian Federation; ^16^ Amur Regional Oncology Center Blagoveshchensk Russian Federation; ^17^ Republican Dispensary of Tatarstan Kazan Russian Federation; ^18^ Novosibirsk Regional Cancer Center Novosibirsk Russian Federation; ^19^ Omsk Regional Cancer Center Omsk Russian Federation; ^20^ Tver Regional Cancer Center Tver Russian Federation; ^21^ Khabarovsk Regional Cancer Center Khabarovsk Russian Federation; ^22^ Department of Oncology and Radiation Therapy V.F. Voyno‐Yasenetsky Krasnoyarsk State Medical University Krasnoyarsk Russia

**Keywords:** avelumab, maintenance therapy, metastatic urothelial carcinoma, real‐world data

## Abstract

**Background:**

This ambispective study was designed to assess the efficacy and safety of avelumab maintenance in a real‐world population of patients with metastatic urothelial cancer (UC).

**Methods:**

Patients with metastatic UC and measurable disease that had not progressed following first‐line platinum‐based chemotherapy were treated with maintenance avelumab (800 mg administered every 2 weeks). The primary endpoint was overall survival (OS).

**Results:**

A total of 110 patients were enrolled. The majority of patients were male (81%), with a median age of 65 years (range, 36–84). The median OS was not reached, with a 1‐year OS rate of 78.7%. The median PFS was 9.5 months (95% CI, 7.8–11.2 months). The ORR to first‐line chemotherapy was 48.2%, and an additional 34.6% of patients responded to avelumab therapy (16 complete and 22 partial responses). Grade 3 adverse events during avelumab therapy were experienced by 11.8% of patients.

**Conclusions:**

These findings demonstrate similar efficacy and safety of avelumab in a real‐world setting when compared to data from pivotal study.

**Trial Registration:** KCRB registry number: RAVE‐Bladder

## 1 Introduction

Over the next 15 years, the incidence of bladder cancer is expected to increase, making it one of the top 5 most common tumors in men [1, 2]. In addition to the rise in overall morbidity, it is plausible to anticipate an increase in the number of patients diagnosed with metastatic disease. The introduction of novel treatments that show enhanced overall survival (OS) outcomes could potentially influence mortality rates associated with bladder cancer [3].

Until recently, the only randomized trial that demonstrated a statistically significant improvement in OS in patients with metastatic urothelial cancer (UC) who had received first‐line chemotherapy and had no disease progression was the Javelin Bladder 100 study [4]. Following the study, the current standard of care for the initial treatment of patients diagnosed with unresectable locally advanced or metastatic UC shifted to platinum‐based chemotherapy with maintenance therapy involving avelumab [5]. The latest update to the study illustrates a median OS of 23.8 months, a median progression‐free survival (PFS) of 5.5 months, and an objective response rate (ORR) to avelumab of 14.3% [6]. With a median follow‐up of 38.0 months, any‐grade adverse events (AEs) occurred in 78.2% of avelumab‐treated patients, including grade ≥ 3 AEs in 19.5% of cases, and these findings affirmed the sustained safety profile of avelumab [6].

It is known that patients with genitourinary malignancies encountered in real‐world practice may diverge from those selected based on the inclusion criteria of clinical trials [7, 8]. Usually, discrepancies may arise due to variations in age, overall performance status, laboratory values, the extent and location of disease spread, as well as the types of therapies administered [9, 10, 11].

This RAVE‐Bladder study was designed to assess the efficacy and safety of avelumab maintenance in a real‐world population of patients with metastatic UC.

## 2 Methods

### 2.1 Study Design and Treatment

The design of this ambispective study included both prospective and retrospective components. In the retrospective part of the study, no more than 30% of patients were permitted for inclusion if they strictly met the eligibility criteria and initiated avelumab treatment no later than 6 months before the study commencement. Inclusion criteria comprised newly diagnosed, histologically confirmed metastatic UC; age ≥ 18 years at diagnosis; measurable disease according to the Response Evaluation Criteria in Solid Tumors (RECIST), version 1.1; and no disease progression (an ongoing response or stable disease) after receiving at least four cycles of first‐line platinum‐based chemotherapy. Patients who had previously received neoadjuvant or adjuvant chemotherapy for nonmetastatic UC were also eligible. Patients involved in clinical trials or treated with other systemic anticancer therapy were excluded. PD‐L1 expression was not a mandatory inclusion criterion.

Following a chemotherapy, avelumab maintenance at a flat dosing of 800 mg administered intravenously every 2 weeks was initiated. Reductions in the dose of avelumab were not allowed. Treatment continued until disease progression, unacceptable toxicity or other criteria for discontinuation occurred.

The RAVE‐Bladder study complied with the rules of the Declaration of Helsinki. The study was approved by the principal investigators and study group. All patients provided written informed consent to treatment with avelumab.

### 2.2 End Points and Assessments

The primary end point was median OS. Secondary end points included 1‐year OS rate, median PFS, ORR, median duration of response and stable disease, and safety.

Disease progression was assessed based on radiological and clinical data, and in addition, change in therapy and death were markers of disease progression. Follow‐up was conducted every 8 (±2) weeks using computed tomography or in some cases magnetic resonance imaging until the confirmed disease progression, date of death, or last visit if alive. Response was measured according to RECIST, version 1.1. AEs were graded according to the National Cancer Institute Common Terminology Criteria for Adverse Events, version 5.0. Switching from first‐line therapy to subsequent‐line therapy has also been studied. Switching to the next line was defined as a change in treatment as a result of disease progression or toxicity.

In some patients, immunohistochemical PD‐L1 expression was evaluated in tumor samples using SP263 clone (Ventana Medical Systems).

### 2.3 Statistical Analysis

The final efficacy and safety analysis included patients who received at least one dose of the avelumab. Descriptive statistics (means, medians, and proportions) were used to describe baseline patient characteristics and treatment regimens. Quantitative data were expressed as mean ± standard deviation, and the Mann–Whitney *U* test was performed as a nonparametric test to analyze variables that were not normally distributed to evaluate the relationships between variables. The survival time was calculated from the day of start of avelumab treatment to the day of death (OS), to the day of disease progression, or to the day of death from any cause (PFS). One‐year survival rate was defined as the proportion of patients in the study still alive at 12 months from the start of treatment. Survival curves were evaluated using the Kaplan–Meier method. The associations between outcomes and clinical and demographic factors were assessed by Kaplan–Meier analyzes and log‐rank comparisons. All *p*‐values were two‐sided, and values of < 0.05 were considered to be statistically significant. Tests assumed a 95% confidence interval (CI). All statistical analyzes were performed using the IBM SPSS Statistics Base v22.0 software (SPSS Inc., Chicago, Illinois, USA).

## 3 Results

### 3.1 Patient and Treatment Characteristics

Between June 2022 and December 2023, a total of 110 patients were recruited from 21 comprehensive cancer centers. Eighty‐nine (80.9%) patients were enrolled prospectively. The baseline characteristics of the patients are outlined in Table 1. The median age was 65 years (range, 36–84), with 35.5% of patients aged > 70 years. The majority of patients were male (*N* = 89, 81%). All patients had confirmed UC, with 62.8% being with high grade. PD‐L1 expression was assessed in 28 (25.5%) cases, with 17.9% testing positive. The primary tumor site was the upper urinary tract in 26 (23.6%) cases and the bladder in 84 (76.4%) cases. Forty‐five (40.9%) patients underwent radical surgery in the history. Sixty‐eight (61.8%) patients had metastatic disease at the time of diagnosis, with 44 (40%) having metastases in two or more sites. The median neutrophil‐to‐lymphocyte ratio was 1.76 (range, 0.55–4.9). Almost all patients (*N* = 101; 91.8%) had chronic or concomitant diseases.

**TABLE 1:** Patient and treatment characteristics.

All patients, *N*	110
Age, years, median (range)	65 (36–84)
Gender, *N* (%)	
Male	89 (81)
Female	21 (19)
Geographic region of Russia, *N* (%)	
European part	74 (67)
Asian part	36 (33)
Histological subtype of bladder cancer, *N* (%)	
Urothelial carcinoma	110 (100)
Grade of tumor, *N* (%)	
High	69 (62.8)
Low	41 (37.2)
Primary tumor site, *N* (%)	
Upper urinary tract	26 (23.6)
Bladder	84 (76.4)
PD‐L1 expression, *N* (%)	
Assessed patients	28 (25.5)
PD‐L1 positive	5 (17.9)
Metastases at time of diagnosis, *N* (%)	
Yes	68 (61.8)
No	42 (38.2)
Visceral metastases, *N* (%)	
Yes	71 (64.5)
No	39 (35.5)
Organs with metastases, *N* (%)	
1	66 (60)
≥ 2	44 (40)
History of surgery, *N* (%)	
Yes	45 (41)
No	65 (59)
First‐line systemic chemotherapy, *N* (%)	
Gemcitabine + cisplatin	51 (46.4)
Gemcitabine + carboplatin	32 (29)
Cisplatin only	19 (17.3)
Carboplatin only	7 (6.4)
ddMVAC	1 (0.9)
Number of chemotherapy cycles, *N* (%)	
4	61 (55.5)
5	8 (7.3)
6	38 (34.5)
7	2 (1.8)
9	1 (0.9)
Response to first‐line chemotherapy, *N* (%)	
Objective response rate	53 (48.2)
Complete response	16 (14.6)
Partial response	37 (33.6)
Stable disease	57 (51.8)

First‐line chemotherapy regimens included gemcitabine + cisplatin, gemcitabine + carboplatin, cisplatin only, carboplatin only, and MVAC in 51 (46.4%), 32 (29%), 19 (17.3%), 7 (6.4%), and 1 (0.9%) patients, respectively. The median number of chemotherapy cycles was 4 (range, 4–9), with 49 (44.5%) patients receiving 5 or more cycles. The ORR to first‐line chemotherapy was 48.2%, including complete responses in 16 (14.6%) patients, partial responses in 37 (33.6%) patients, and stable disease in 57 (51.8%) patients.

The median number of avelumab infusions was 19 (range, 2–48). The most common reason for permanent discontinuation of treatment was progressive disease in 65 (59.1%) patients. Three (2.7%) patients discontinued treatment due to AEs.

### 3.2 Efficacy and Safety

**FIGURE 1:**
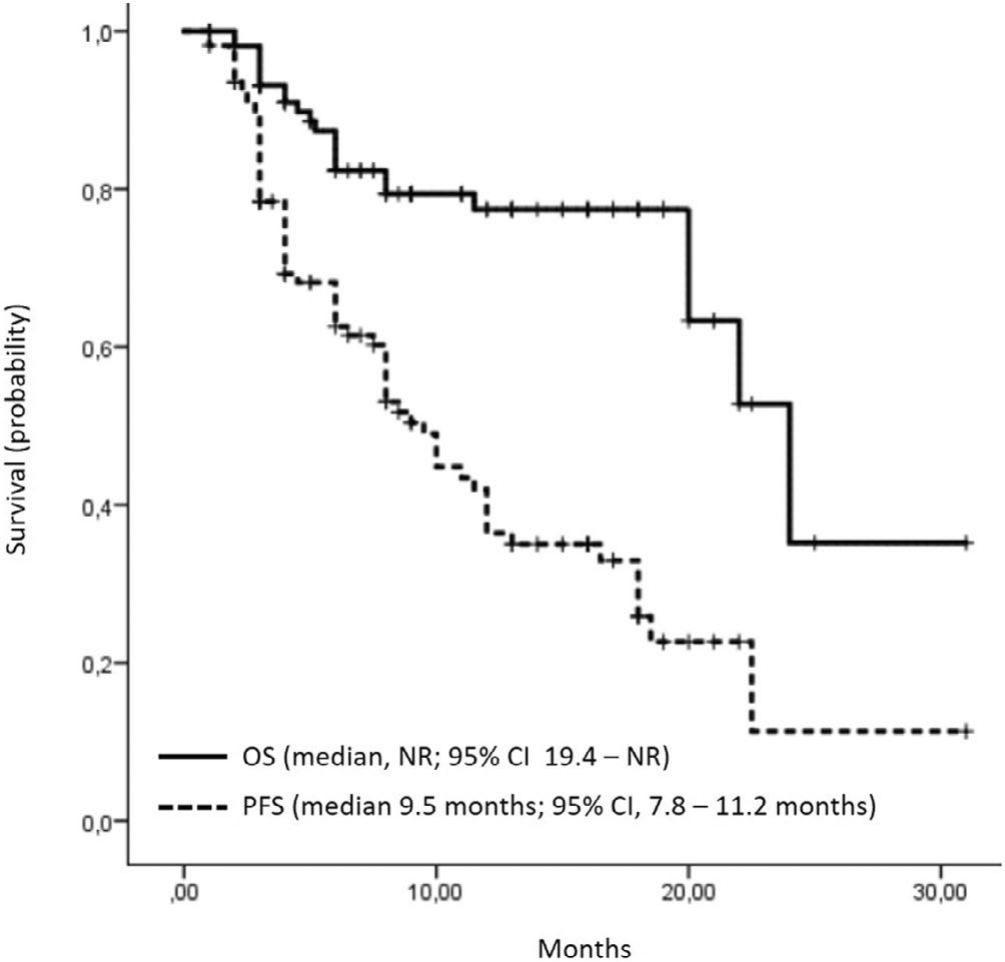
Overall and progression‐free survival (Kaplan–Maier curves).

At a median follow‐up of 11.9 months, the median OS was not reached, with 85 (77.3%) patients still alive (Figure 1). The 1‐year OS rate was 78.7%. Patients who did not experience disease progression at the time of the last follow‐up demonstrated a significantly longer OS compared to patients with disease progression (median not reached vs. 22.0 months, *p* = 0.001).

At data cutoff on April 30, 2024, the median PFS was 9.5 months (95% CI, 7.8–11.2 months). In the univariate analysis, there was no significant difference in median PFS (12.0 vs. 8.0 months; *p* = 0.42) between the cohorts of patients receiving combination chemotherapy (gemcitabine and cisplatin or carboplatin) and those receiving platinum monotherapy (cisplatin or carboplatin alone). According to Russian guidelines (RUSSCO), platinum monotherapy is not a treatment option; however, platinum monotherapy was prescribed due to interruptions in gemcitabine supplies in two centers during treatment of these patients.

In the cohort of patients assessed for PD‐L1 expression, the median PFS was numerically better in PD‐L1‐positive patients (≥ 25% of positive cells) compared to PD‐L1‐negative patients (16.7 vs. 7.18 months); however, these differences did not reach statistical significance (*p* = 0.194). The analysis did not reveal significant differences in PFS according to various patient and treatment characteristics, including age (< 70 vs. ≥ 70, *p* = 0.79), gender (male vs. female, *p* = 0.44), location of the primary tumor (upper urinary tract vs. bladder, *p* = 0.44), grade of tumors (low vs. high, *p* = 0.099), presence of metastases at diagnosis (yes vs. no, *p* = 0.79), history of surgery (yes vs. no, *p* = 0.82), organs with metastases (visceral vs. nonvisceral, *p* = 0.05), number of cycles of first‐line chemotherapy (4 vs. ≥ 5, *p* = 0.093), and response to first‐line therapy (response vs. stable disease, *p* = 0.302). There was a trend suggesting that patients with neutrophil‐to‐lymphocyte ratio below the median had better results of PFS, with a median of 11 months compared to 6 months for those with neutrophil‐to‐lymphocyte ratio above the median (*p* = 0.055).

**FIGURE 2:**
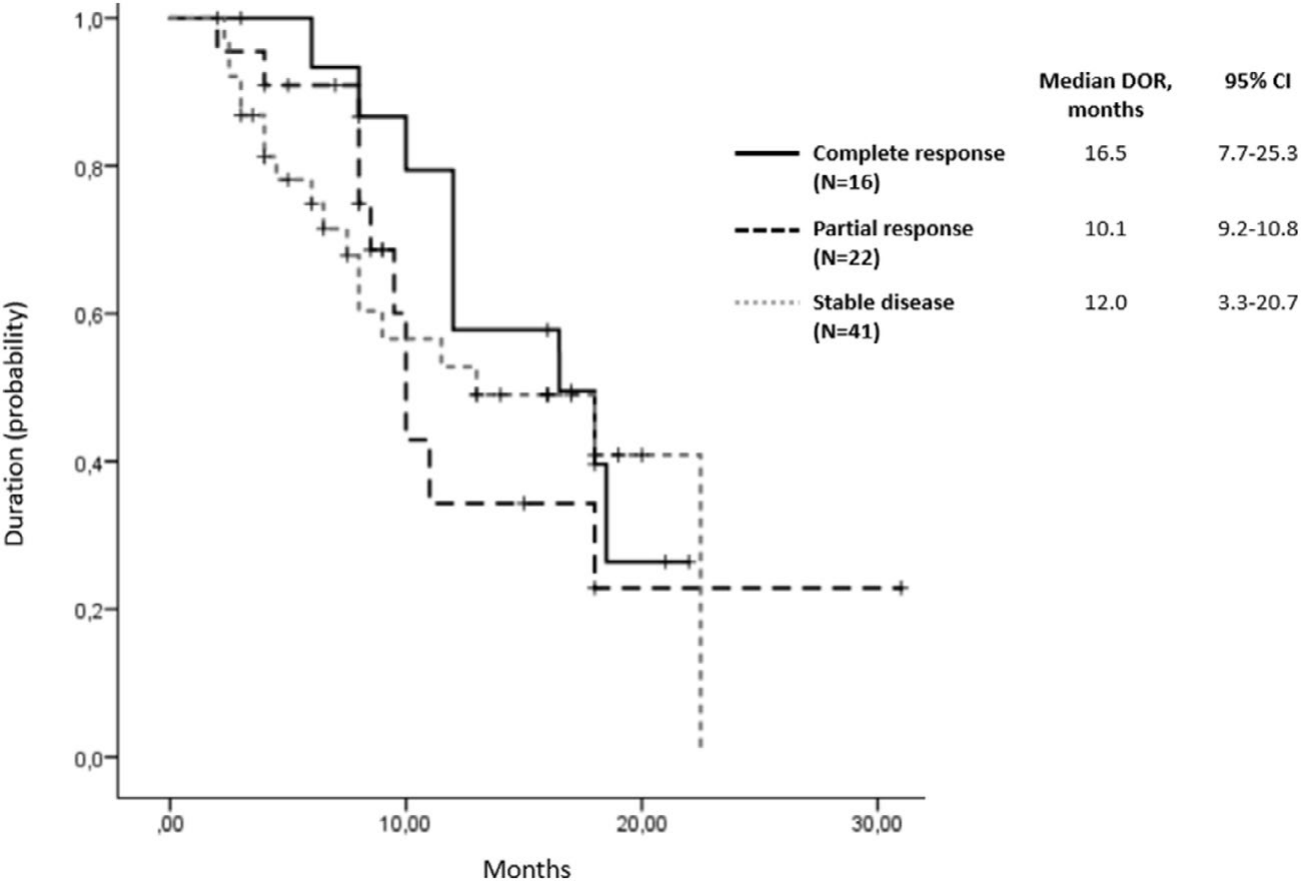
Duration of response in assessed patients.

Response rate to avelumab therapy was evaluated in 97 (88.2%) patients. The rate of assessed objective response was 39.2% (95% CI, 36–42). Among them, 16 (16.5%) patients achieved complete responses, 22 (22.7%) achieved partial responses, 41 (42.3%) had stable disease, and 18 (18.6%) experienced progressive disease. Over the 11 months of follow‐up, a 30% increase in response rate was observed. Complete responses demonstrated the longest duration (median 16.5 months, 95% CI, 7.7–25.3; Figure 2). At data cutoff, a total of 44 patients (40%) were still receiving trial treatment. In patients who discontinued study therapy because of progressive disease, 19 of 65 (29.2%) received a subsequent anticancer drug therapy, including a PD‐1/PD‐L1 inhibitor in 3 (4.6%).

In all treated patients, any‐grade TRAEs occurred in 89 (80.9%) cases, including grade ≥ 3 TRAEs in 13 (11.8%; Table 2). The most serious AEs were adrenal insufficiency, arthralgia, pulmonary embolism, and proteinuria.

**TABLE 2:** Grade ≥ 3 adverse events.

TRAE	*N* (%)
All grade ≥ 3 adverse events	13 (11.8)
Fatigue	2 (1.8)
Hypothyroidism	2 (1.8)
Vomiting	2 (1.8)
Anemia	1 (0.9)
Neutropenia	1 (0.9)
Platelet count decreased	1 (0.9)
Adrenal insufficiency	1 (0.9)
Arthralgia	1 (0.9)
Pulmonary embolism	1 (0.9)
Infusion‐related reaction	1 (0.9)

## 4 Discussion

In the RAVE‐Bladder real‐world study, which involved over 100 patients with metastatic UC who had not experienced progression following standard chemotherapy, we evaluated the efficacy and safety of avelumab maintenance therapy. Overall, our study findings align with the data from the Javelin Bladder 100 prospective study [6]. Specifically, the 1‐year OS rate was 78.7% in the RAVE‐Bladder study compared to 71.3% in the Javelin Bladder 100 trial. In real‐world practice, the response rate to avelumab increased progressively, and with a median follow‐up of approximately a year, one‐third of all patients responded to the therapy. Furthermore, we demonstrated that complete responses to avelumab therapy exhibited durability, with a median duration of 16.5 months. The period of disease control was longer than observed in the prospective randomized trial, as evidenced by a median PFS of 9.5 months compared to 5.5 months, respectively. This extended disease control period may have influenced the median OS, which was not reached in the subgroup of patients who had not experienced disease progression at the time of data analysis, whereas it was 22 months in the subgroup of patients with progressive disease.

It is important to note that the baseline characteristics of patients in real‐world settings were comparable to those of the cohort in the Javelin Bladder 100 trial; however, the real‐world population was more heterogeneous due to various factors. For instance, there was a higher proportion of patients with visceral metastases (64.5% vs. 54.6%), patients who received first‐line carboplatin or cisplatin as single agents (23.7% vs. 0%), and patients who underwent only 4 cycles of prior chemotherapy (55.5% vs. 36.3%). Real‐world practice often presents with unfavorable prognostic factors, such as a significant number of patients with high‐grade tumors (62.8%) and disease spread to more than two organs (40%). Despite these challenges, subgroup analysis from our study affirmed that avelumab first‐line maintenance therapy is suitable for patients with a diverse range of characteristics. We particularly emphasize that no significant differences in PFS were observed among patients receiving different types of chemotherapy.

One potential predictor of avelumab effectiveness could be the neutrophil‐to‐lymphocyte ratio, as highlighted in several prior studies [12, 13, 14]. In our study, the median PFS differed by 1.8‐fold between patients with ratio below the median and those above the median. Interestingly, baseline median neutrophil‐to‐lymphocyte ratio in RAVE‐Bladder and other real‐world studies tends to be lower compared to that in randomized trials [15]. However, it should be noted that the limited number of patients in these subgroups prevents us from drawing definitive conclusions.

In the RAVE‐Bladder study, all patients received avelumab at a flat dose of 800 mg, which differs from the dosing regimen used in the Javelin Bladder 100 study (10 mg/kg). Despite this difference, safety indicators were not affected. The incidence of all avelumab‐related AEs was comparable between the two studies (80.9% and 78.2%). Moreover, the incidence of grade ≥ 3 AEs was even lower in the RAVE‐Bladder study (11.8%) compared to that in the Javelin Bladder 100 study (19.5%), although this difference may be attributed to the twofold shorter follow‐up period. Notably, treatment interruption due to poor tolerability occurred in only three patients.

Several noninterventional studies have corroborated the efficacy and safety of avelumab as the first‐line maintenance therapy in patients with advanced UC. The largest among them, the ambispective AVENANCE study, involved 595 patients, with 82.5% being men and 91.9% having metastatic disease, predominantly with visceral metastases (84.8%). Bladder was the primary tumor site in 74.9% of cases. First‐line chemotherapy regimens included carboplatin + gemcitabine in 61.5% patients, cisplatin + gemcitabine in 27.9% patients, and ddMVAC in 4.2% patients. After a median follow‐up of 26.3 months, the median OS from the start of avelumab was 21.3 months, with a 1‐year OS rate of 66.52%. The median PFS was 5.7 months. TRAEs of any grade were reported in 59.1% of patients, with 6.2% being serious. Despite differences in the proportion of patients receiving first‐line carboplatin/cisplatin + gemcitabine in AVENANCE and potential variations in patient populations between studies, the efficacy and safety results in the RAVE‐Bladder study were comparable to those reported in the AVENANCE trial. A comparison of results among different studies involving more than 100 patients treated with avelumab is provided in Table 3.

**TABLE 3:** Results of clinical trials with inclusion of > 100 avelumab‐treated patients.

	Number of avelumab‐treated patients	Design	OS, median, months	1‐year OS rate, %	PFS, median, months	ORR, %	TRAEs, all grades (grade ≥ 3), %
Javelin Bladder 100 [4, 6]	350	Prospective randomized Phase 3	23.8	71.3	5.5	14.3	78.2 (19.5)
AVENANCE [16]	595	Ambispective	21.3	66.52	5.7	—	59.1
READY [17]	464	Prospective, noninterventional compassionate use program	NR	69.2	8.1	—	(7.1)
PATRIOT II [18]	160	Retrospective	24.4	75.7	5.4	—	38.8
Bakaloudi et al. [19]	108	Retrospective	NR	72.5	9.6	28.7	—
RAVE‐Bladder	110	Ambispective	NR	78.7	9.5	39.2	80.9 (11.8)

Abbreviations: OS: overall survival; PFS: progression‐free survival; ORR: objective response rate; TRAEs: treatment‐related adverse events; NR: non reached.

Maintenance therapy with avelumab has shown significant improvements in PFS and OS in real‐world practice compared to the era when chemotherapy alone was the standard first‐line treatment for metastatic UC. For instance, in a Danish registry including 952 patients from 2017 to 2019, the median OS was 11.7 months [20]. Similarly, in a German registry with 435 patients included in 2016 [21], the median OS was 16.1 months. In the US registry covering 1811 patients from 2011 to 2017 [22], the median OS was 12.7 months. Moreover, in developing countries where fewer patients received platinum‐based chemotherapy, these rates were even lower. In the Russian URRU registry study involving 246 patients between 2017 and 2018, the median OS was 7.0 months, with a 1‐year OS rate of 34% [23, 24]. Likewise, in a similar OSURK registry study conducted in Kazakhstan, which enrolled 480 patients from 2017 to 2018, the median OS was 7.3 months, with a 1‐year OS rate of 31% [8]. Hence, the findings from the RAVE‐Bladder study indicate the need for further integration of modern therapies into real‐world practice in order to increase the OS and PFS in patients with previously untreated metastatic UC.

In conclusion, these results of the RAVE‐Bladder study showed similar efficacy and safety of avelumab in real‐world setting compared to data from other prospective and retrospective studies. Ongoing follow‐up continues to provide additional insights.

## Author Contributions

**Ilya Tsimafeyeu:** conceptualization, methodology, software, data curation, formal analysis, validation, investigation, writing – original draft, writing – review and editing, project administration. **Yana Gridneva:** conceptualization, methodology, investigation, writing – review and editing. **Alexander Sultanbaev:** investigation, writing – review and editing. **Yulia Anzhiganova:** conceptualization, investigation, methodology, writing – review and editing. **Mark Gluzman:** writing – review and editing, investigation. **Anastasia Mochalova:** investigation, writing – review and editing. **Alexey Shkurat:** investigation, writing – review and editing. **Edgar Israelyan:** investigation, writing – review and editing. **Elvira Parsadanova:** investigation, writing – review and editing. **Yulia Murzina:** investigation, writing – review and editing. **Aleksei Ivanov:** investigation, writing – review and editing. **Alexey Kalpinskiy:** investigation, writing – review and editing. **Olesia Stativko:** investigation, writing – review and editing. **Vladislav Petkau:** investigation, writing – review and editing. **Elena Karabina:** investigation, writing – review and editing. **Artyom Kеln:** investigation, writing – review and editing. **Natalya Tovbik:** investigation, writing – review and editing. **Sufia Safina:** investigation, writing – review and editing. **Maria Turganova:** investigation, writing – review and editing. **Evgeny Kopyltsov:** investigation, writing – review and editing. **Varvara Bragina:** investigation, writing – review and editing. **Olga Novikova:** investigation, writing – review and editing. **Andrei Lebedinets:** investigation, writing – review and editing. **Vladislav Vodolazskiy:** investigation, writing – review and editing. **Alexey Rumyantsev:** investigation, writing – review and editing. **Ruslan Zukov:** investigation, supervision, writing – review and editing. **Ilya Pokataev:** investigation, supervision, writing – review and editing. **Rashida Orlova:** investigation, supervision, writing – review and editing. **Maria Volkova:** conceptualization, investigation, methodology, writing – review and editing, supervision, data curation.

## Ethics Statement

Study procedures were in accordance with ethical guidelines for human subject's research and approved by the BUCARE Review Board (Protocol #KCRB01012022).

## Consent

All patients provided written informed consent to treatment with avelumab.

## Conflicts of Interest

The authors declare no conflicts of interest.

## Data Availability

The datasets generated during and/or analyzed during the current study are available from the corresponding author on reasonable request.
